# Efficacy of Serotonin and Dopamine Activity Modulators in the Treatment of Negative Symptoms in Schizophrenia: A Rapid Review

**DOI:** 10.3390/biomedicines11030921

**Published:** 2023-03-16

**Authors:** Claudio Brasso, Gianluca Colli, Rodolfo Sgro, Silvio Bellino, Paola Bozzatello, Cristiana Montemagni, Vincenzo Villari, Paola Rocca

**Affiliations:** 1 Department of Neuroscience “Rita Levi Montalcini”, University of Turin, 10124 Torino, Italy; 2 Psychiatric Emergency Service, Department of Neuroscience and Mental Health, Azienda Ospedaliero-Universitaria “Città della Salute e della Scienza di Torino”, 10126 Turin, Italy

**Keywords:** schizophrenia, schizophrenia spectrum disorders, negative symptoms, serotonin-dopamine activity modulators, SDAMs, aripiprazole, cariprazine, brexpiprazole, lumateperone, antipsychotic

## Abstract

Schizophrenia is among the fifteen most disabling diseases worldwide. Negative symptoms (NS) are highly prevalent in schizophrenia, negatively affect the functional outcome of the disorder, and their treatment is difficult and rarely specifically investigated. Serotonin-dopamine activity modulators (SDAMs), of which aripiprazole, cariprazine, brexpiprazole, and lumateperone were approved for schizophrenia treatment, represent a possible therapy to reduce NS. The aim of this rapid review is to summarize the evidence on this topic to make it readily available for psychiatrists treating NS and for further research. We searched the PubMed database for original studies using SDAM, aripiprazole, cariprazine, brexpiprazole, lumateperone, schizophrenia, and NS as keywords. We included four mega-analyses, eight meta-analyses, two post hoc analyses, and 20 clinical trials. Aripiprazole, cariprazine, and brexpiprazole were more effective than placebo in reducing NS. Only six studies compared SDAMs with other classes of antipsychotics, demonstrating a superiority in the treatment of NS mainly for cariprazine. The lack of specific research and various methodological issues, related to the study population and the assessment of NS, may have led to these partial results. Here, we highlight the need to conduct new methodologically robust investigations with head-to-head treatment comparisons and long-term observational studies on homogeneous groups of patients evaluating persistent NS with first- and second-generation scales, namely the Brief Negative Symptom Scale and the Clinical Assessment Interview for Negative Symptoms. This rapid review can expand research on NS therapeutic strategies in schizophrenia, which is fundamental for the long-term improvement of patients’ quality of life.

## 1. Introduction

Schizophrenia (SZ) is the 12th leading cause of disability worldwide, with an estimated burden of disease of 13.4 million years of life lived with disability [1]. Therefore, an effective and efficient treatment of this disorder may have a positive impact on the well-being and quality of life of patients and their caregivers [2]. In this section, we briefly present the neurobiological correlates of the disorder, negative symptoms (NS), and the main serotonin and dopamine activity modulators (SDAMs).

From a pathophysiological point of view, SZ is considered a neurodevelopmental disease with a neuroprogressive component consisting of a reduction in the density of the dendritic spines and a dysfunction in the synaptic homeostasis and glial cells without gliosis and neuronal necrosis [3,4,5,6,7,8,9,10,11,12]. Many factors, partly shared with other neuropsychiatric diseases, may contribute to these neurobiological underpinnings of the disorders. 

Regarding the alteration of correct neurodevelopment, there were identified several causes related to intracellular alteration of neurons, such as the case of Disrupted in schizophrenia 1 (DISC1), encoded by the DISC1 gene, which is a protein that plays a role in presynaptic regulation of dopamine, and its alterations increase the risk of SZ [13]. DISC1 plays various roles in several cellular functions, including mitochondrial transport, fission, and fusion [14]. In mouse models, an altered form of DISC1 is correlated with neuroanatomical changes, including displaced dentate granule neurons, altered axonal targeting, reduced dendrite growth, and dendritic spine density. Moreover, mouse models with altered forms of DISC1 were associated with behavioral alterations including impairment of working memory [15], but also behaviors resembling cognitive, psychotic, and negative symptoms of SZ [16]. 

The neuroprogressive component of SZ is thought to be the outcome of several interrelated processes including mitochondrial impairment, oxidative stress responses, and activation of immune responses, leading to chronic low-grade inflammation [17]. In particular, mitochondrial functioning contributes to undermining the normal neural connectivity in the brain [18,19]. A meta-analysis on this topic reported abnormal expression patterns of mitochondrial respiratory complex I in frontal cortex, cerebellum, and striatum [20] post-mortem samples of patients with SZ. 

An alteration of the kynurenine pathway of tryptophan degradation may represent another factor contributing to the neuroprogression in SZ. In particular, higher levels of kynurenine and kynurenic acid in plasma, cerebrospinal fluid, brain tissue, and saliva were found in patients with SZ as compared to healthy controls [21], suggesting that the kynurenine pathway is more active in patients with SZ. This higher concentration of kynurenic acid was related to the cognitive deficits of SZ [22].

Neural correlates related to these alterations are studied in vivo with different neuroimaging techniques, such as structural and functional magnetic resonance imaging (MRI), positron emission tomography (PET), and electroencephalography (EEG). Structural MRI revealed alteration of the gray and white matter of the brain in patients with SZ as compared to healthy controls. In particular, the reduction in gray matter was more frequent in the medial prefrontal cortices, the inferior, medial and superior frontal and temporal gyri, the supplementary motor area, the angular gyri (temporo-parietal junction), the anterior cingulate and insular cortices, the parahippocampal cortices, the amygdalae, the thalami, and the caudate nuclei [23,24,25,26,27,28]. Alterations of the white matter were found mostly in long projection fibers, callosal and commissural fibers, parts of motor descending fibers, and frontotemporal-limbic pathways [29]. At a functional level, alterations were found both during resting state and during specific tasks. A resting-state dysconnectivity was observed in the limbic, frontoparietal executive, default mode, and salience networks. These networks involve the simultaneous activity of cerebral areas with reduced gray matter in SZ, such as the insula, lateral postcentral cortex, striatum, and thalamus [30]. An altered dynamic functional connectivity during resting state was also found in the cuneus [31]. As to task-related functional MRI, many studies found a decreased activation of the dorsomedial prefrontal cortex, the supplementary motor area, and the right inferior frontal gyrus during tasks involving neurocognitive processes and of the right angular gyrus during social-cognitive tasks [24]. Concerning EEG, abnormalities were found in alpha, theta and gamma activity, as well as in mismatch negativity and P300 waves, and were associated with impairments in different neurocognitive domains such as attention, working memory, visual and verbal learning, and executive functioning [32]. Finally, PET studies assessing brain glucose metabolism with 18-fluorodeoxyglucose evidenced a hypometabolism in the frontal cortex without significant alterations in other brain regions [33].

People with SZ can suffer from both positive symptoms, i.e., hallucinations and delusions, and negative symptoms (NS), including deficits in affect, speech, motivation, and sociality [34]. Clinically relevant NS are extremely prevalent (50–90%) in individuals with SZ and significantly limit their social and occupational functioning [35,36,37,38,39,40,41,42,43]. In addition, higher NS are associated with a poorer quality of life, for both patients and their caregivers, [44] and higher health care costs and resource utilization [45].

The term NS was first employed in neurology in the first half of the 19th century to indicate motor and cognitive deficits. In the same period, Haslam [46] (1809) described a thymic deficit in young people characterized by blunted sensitivity and affective indifference. In 1865, Griesinger [47] coined the term “fixed affective madness” for patients that were inaccessible to emotions and anergic. In 1919, Kraepelin [48] described irreversible and progressive deficits, in particular affective blunting and loss of mastery over volition, in patients with the syndrome, which he called dementia praecox. In 1911, Bleuler [49] introduced the term schizophrenia and in 1950 suggested that affective blunting and emotional withdrawal were fundamental symptoms of the disorder. Accordingly, in 1954, Minkowski [50] suggested that instinctive-affective deficit was a primary characteristic of SZ, while loss of reality was a secondary one. Between the late 1960s and the late 1980s, different authors focused on the "negative dimension" of the disorder [51,52,53], and in 1982 the first scale for the assessment of NS (SANS) was proposed by Andreasen et al. [54]. Over the next two decades, additional tools were developed, leading to various constructs belonging to NS and consequently to a lack of scientific consensus on this topic. In 2005, a meeting sponsored by the United States National Institute for Mental Health partially solved this problem, leading to the construct of NS that predominates to this day [55,56]. According to the consensus statement from the meeting, NS were divided into five domains, namely avolition, asociality, anhedonia, alogia, and blunted affect [56,57]. These domains were defined as a reduction in the initiation and persistence of goal-directed activities (avolition), in the drive to form and maintain relationships with others (asociality), in the experience of pleasure (anhedonia), in the expression of emotions (blunted affect), and in the number of words spoken and in spontaneous elaboration (alogia) [37,56,57,58]. These five domains were grouped into two factors, i.e., experiential NS, consisting of avolition, asociality, and anhedonia, and expressive NS, including alogia and blunted affect [37,38,55,56,57,58,59]. Even if the five domains are highly interrelated, many studies demonstrated the construct validity of both the five domains and two factor models of NS [37,60,61,62,63]. The partial independence of these five or two constructs has an important clinical relevance, as different constructs might show different responses to treatments [55]. Hypothetically, expressive NS, but not experiential ones, might improve after a specific psychosocial intervention. If those two factors were not evaluated separately, this specific effect might be overlooked. 

A potentially useful option to test the efficacy of treatments in reducing NS consists of a longitudinal evaluation of the experiential and expressive factors with specific items of first-generation scales complemented by second-generation ones [58]. The selection of specific items of first-generation scales to assess core NS is indicated to exclude potential confounding factors belonging to other symptoms, such as depressive, cognitive, and positive ones [58]. The concomitant use of second-generation scales is suggested, as they cover all five domains of NS and take into consideration also patients’ subjective experiences without including confounding factors. These kinds of instruments include the Brief Negative Symptom Scale (BNSS) [64] and the Clinical Assessment Interview for Negative Symptoms (CAINS) [65].

A further clinically useful classification of NS consists of the distinction between primary and secondary NS [37,53,66,67,68]. The former are supposed to derive directly from SZ, while the latter are caused by other factors such as positive and depressive symptoms, medication adverse effects, isolation, and substance abuse [37,53,66,67,68]. This distinction is clinically relevant, as secondary NS might benefit from treating the underlying causes. A useful strategy to handle secondary NS consists of multiple longitudinal evaluations of positive, depressive, and extrapyramidal symptoms to assess their relationship with the fluctuation of NS [37,38,53,58,66,67]. Although effective, this kind of approach is time- and cost-consuming, therefore, inefficient in clinical trials aimed at evaluating the efficacy of the treatments in reducing primary NS. A possible solution to this problem was proposed by the above-mentioned consensus statement on NS day [55] that suggested testing the efficacy of a treatment on persistent NS (PNS), i.e., NS that persist over time, including periods of clinical stability, despite adequate antipsychotic drug treatment [37,55,58,59,69]. One of the most recent operational definitions of PNS is the one reported by the European Psychiatric Association (EPA) guidance on the assessment of NS in SZ [58]. This guidance defined PNS as the presence of at least three moderate or two moderately severe NS that persist for at least six months without moderately severe, severe, or very severe positive symptoms, clinically significant depression, and parkinsonism [37,55,58,59,69]. However, as requested by regulatory agencies, two other constructs have been used in clinical trials: predominant NS and prominent NS. The former refers to a prevalence of NS over positive ones and the latter to the presence of a consistent burden of moderately severe or severe NS. None of the constructs take into account the persistence over time of NS. In addition, contrary to PNS, both predominant and prominent NS do not show construct validity and include a mixture of primary and secondary NS likely to fluctuate over time and possibly confound the results of clinical trials [58].

A recent meta-analysis of the MRI brain alterations of patients with PNS found that structural abnormalities were mainly located in bilateral insulae, medial frontal gyri, anterior cingulate gyri, left amygdala, superior temporal gyrus, inferior frontal gyrus, cingulate gyrus, and middle temporal gyrus, while functional alterations were concentrated in the thalamocortical and default mode networks [70]. These results are only partly consistent with the current pathophysiological models of experiential NS [71] that suggest a dysfunction in communication between the subcortical and cortical areas. In particular, they attribute a pivotal role to the dopaminergic neurons of two subcortical areas, namely the ventral tegmental area and the pars compacta of the substantia nigra [72,73,74,75,76,77]. These neurons may activate other subcortical regions such as the nucleus accumbens and the dorsal striatum or the dorsolateral and ventromedial prefrontal cortices [72,73,74,75,76,77]. A dysfunction of these circuits might lead to a lesser degree of motivation and anticipatory pleasure, thus facilitating avolition, asociality, and anhedonia [38,71,78]. Pathophysiological mechanisms of expressive NS have been poorly investigated [71]. These NS were related to neurocognitive and social cognition deficits and to soft neurological signs [38,71,79,80,81].

Since the first use of chlorpromazine to treat SZ in 1952 [82], only a few studies have focused on the efficacy of antipsychotic drugs in reducing NS. Most of them were published in the last three decades and focused on second-generation compounds, since first-generation antipsychotics showed little or no efficacy in the treatment of NS [83,84,85,86]. When compared to placebo or first-generation antipsychotics, some second-generation antipsychotics showed little or moderate efficacy in the treatment of NS. These drugs were amisulpride, clozapine, olanzapine, and risperidone [87,88,89,90,91]. 

Starting from 1987, the year of the discovery of aripiprazole, a new type of antipsychotic drug has been developed [92]. Functionally, they can be referred to serotonin-dopamine activity modulators (SDAMs) and consist of four drugs approved by the United States Food and Drug Administration (FDA) for the treatment of SZ, namely aripiprazole, brexpiprazole, cariprazine, and lumateperone [93]. The first drug approved by the FDA was aripiprazole in December 2002, and the last one was lumateperone in December 2019. SDAMs differ from previous antipsychotic drugs, all of which were D2 dopamine receptor antagonists, in that these compounds are partial agonists of D2 dopamine receptors [94]. Due to this partial agonist activity, when binding to the D2 dopamine receptor in its G-protein-coupled state, these drugs exert a receptor-blocking action in the presence of excess dopamine, and conversely stimulate the receptor when there is no excessive dopamine, thus playing the role of a stabilizer [95]. This partial agonist activity at D2 dopamine receptors is hypothesized to mitigate the overactive dopamine system in striatal regions but increase activity in hypodopaminergic areas such as prefrontal cortex, which may improve NS [96]. Each of the four abovementioned antipsychotic drugs showed specific pharmacodynamics; however, they share a similar mechanism of action, as they modulate simultaneously the serotoninergic and dopaminergic systems, hence the name SDAMs [97,98].

Focusing on the specificity of each SDAM, aripiprazole has very high binding affinities with dopamine D2, D3, and serotonin 5-HT 2B receptors; high binding affinities with serotonin 5-HT 1A and 5-HT 2A receptors; and moderate binding affinities with serotonin 5-HT 2C, 5-HT 7 receptors, dopamine D4 receptors, adrenergic α 2C, α 1B and α 1A receptors, and histamine H1 receptors [99]. Aripiprazole is distinguished from earlier antipsychotics by its partial agonist activity at D2, D3, 5-HT 1A receptor targets [100]; intrinsic activity is 60%, compared to 28%, and 73% for dopamine and serotonin, respectively [101]. 

Brexpiprazole has a greater affinity for D2 receptors and a lesser affinity for D3 receptors compared to both aripiprazole and cariprazine. It also has a higher affinity for D1 and D4 receptors compared to aripiprazole. It acts as a partial agonist of 5HT1A receptors and as an antagonist of 5-HT2A and α1 receptors [102,103]. It binds with higher affinity to serotonin, alpha-adrenergic, and histamine receptors than aripiprazole and cariprazine, except for 5HT2C, for which it has lesser affinity [104,105].

Cariprazine acts as a partial agonist at the D3 and D2 receptors with very high affinity and as an antagonist with high affinity at the 5-HT 2B serotonin receptor. A 10-fold higher binding affinity has been shown for D3 versus D2 receptors in in vitro studies. Compared to aripiprazole, cariprazine has similar intrinsic agonist activity on D2 receptors, but higher intrinsic agonist activity for D3 receptors [106]. This differentiates cariprazine not only from aripiprazole but from most other antipsychotics, which have a low affinity for the D3 receptor. Some authors speculated that this higher affinity for D3 receptors compared to D2 receptors could be effective for negative and cognitive symptoms [90]. Moreover, cariprazine has moderate affinity as a partial agonist for 5-HT 1A receptor, moderate affinity as an antagonist for 5-HT 2A and H1 receptors, and low affinity for 5-HT 2C and *α*1A-adrenergic receptors [106].

Finally, lumateperone has a very high affinity for 5-HT2A serotonin receptor, a moderate affinity for D2 and D3 dopamine receptors and **α**1 and **α**2 noradrenaline receptors, and a low affinity for M1, M3, M4, and 5HT2C receptors. The affinity for 5-HT2A receptors goes over 60-fold compared to the affinity for dopamine receptors, and, at clinical antipsychotic doses, nearly 100% of 5HT2 receptors are saturated by the drug [107]. In vitro studies found that lumateperone acts as an antagonist at postsynaptic D2 receptors [108], but it is still unclear if it acts as an agonist or an antagonist on presynaptic D2 receptors [109]. Moreover, lumateperone can increase the *N*-methyl-d-aspartate (NMDA) receptor activity in mesolimbic regions [110], helping the regulation of the glutamatergic pathways compromised in SZ [111]. It was found that lumateperone indirectly modulates glutamatergic neurotransmission by activating dopamine D1 receptor, subsequently increasing the tyrosine phosphorylation of GluN2B-type *N*-methyl-d-aspartate (NMDA) receptors in mesolimbic/mesocortical dopamine systems [108]. This action should indirectly increase the number of NMDA channels into the membrane of prefrontal neurons, enhancing glutamatergic signaling [112]. Based on the pharmacodynamic profile, particularly on the action on the glutamatergic system, lumateperone might be effective for negative symptoms and cognitive deficits [111]. 

The pharmacodynamics of each SDAM are summarized in Table 1.

### Aims and Rationale

The current literature on the effects of these SDAMs on NS is relatively scarce and fragmentary; therefore, the aim of this rapid review is to summarize the evidence on this topic in order to be make it readily available for psychiatrists in their clinical practice and for researchers to design further studies on the efficacy of these drugs in reducing NS.

This rapid review may enhance the expansion of a specific topic within the narrow field of NS therapeutic strategies in schizophrenia spectrum disorders, which is of fundamental importance to the long-term improvement of patients’ lives.

## 2. Methods

A rapid review is a form of knowledge synthesis that accelerates the process of conducting a traditional systematic review by streamlining various methods to produce evidence for end-users in a resource-efficient manner [115,116]. The end-users of the current rapid review are psychiatrists treating patients with NS and researchers who aim to add evidence on the efficacy of SDAMs in the treatment of NS. The choice of this specific type of review depends on some factors that make it a suitable form of synthesis according to the current guidelines [115,117,118,119]. Those factors are:

i. constraints of rapid review methods (e.g., limited search) will provide sufficient information and be credible for end-users;
ii. the review has a narrow, well-defined scope (e.g., limited population, new drugs);
iii. the amount of evidence on the topic chosen is small;
iv. the evidence to summarize is limited in terms of years of interest;
v. the outcome (i.e., reduction of NS) is relevant to clinicians and patients [115,117,118,119].

### 2.1. Setting the Research Question and Eligibility Criteria

The research question was to evaluate the efficacy of SDAMs in the treatment of NS. To define the research question, we followed the patient population, intervention, comparator, outcome, timing, and setting (PICOTS) framework [120,121,122,123,124,125]. We chose the following PICOTS: adult people with schizophrenia spectrum disorder, treatment with SDAMs, placebo or other antipsychotics, 4 to 52 weeks of follow-up, and psychiatric care facilities.

To set eligibility criteria, we followed the Cochrane evidence-informed guidance to conduct rapid reviews [115]. In detail, we clearly defined the PICOTS and limited the number of interventions to the four SDAMs approved by the FDA for the treatment of SZ, of comparators to placebo or other antipsychotics, and of clinically relevant outcomes to the reduction of NS. We considered as restriction date the day we performed the research for the current review: 9 January 2023. We limited the publication language to English, also included systematic reviews and placed emphasis on higher quality study designs (e.g., systematic reviews and randomized controlled trials). We applied a stepwise approach to study design inclusion as follows: for records about aripiprazole, we included all mega- and meta-analyses and original articles that were not within the mega- and meta-analyses included. For the other three drugs, we included all kinds of contributions.

### 2.2. Search Terms and Electronic Searches

G.C. and R.S. searched PubMed database using the following search strings: ((aripiprazole) OR (cariprazine) OR (brexpiprazole) OR (lumateperone)) AND ((schizophrenia) OR (first episode psychosis) OR (schizophrenia spectrum disorder) OR (schizoaffective disorder) OR (schizophreniform disorder) OR (brief psychotic disorder)) AND (negative symptoms) with a date limit of 9 January 2023. All kinds of articles were included in the search and submitted to retrieval.

### 2.3. Screening and Selection Process

Following the Cochrane evidence-informed guidance to conduct rapid reviews [115], we first screened the title and abstract of the records. C.B., G.C., and R.S. evaluated the same 40 abstracts to calibrate their decision-making process. About 30% of the abstracts were screened by two among C.B., G.C., and R.S. Conflicts were resolved with the opinion of the third researcher. G.C. screened the remaining abstracts, while R.S. screened all excluded abstracts. C.B. resolved conflicts in the title and abstract screening process. C.B., G.C., and R.S. evaluated the same 10 full texts to calibrate their decision-making process. R.S. screened the remaining full texts, while G.C. screened all excluded abstracts. C.B. resolved conflicts in the full-text screening process.

We excluded articles not relevant to our review, i.e., articles written in languages other than English, on drugs other than SDAMs, without negative symptoms change among outcomes, pharmacological studies, in vitro studies, case reports, case series, and animal model studies.

**Figure 1:**
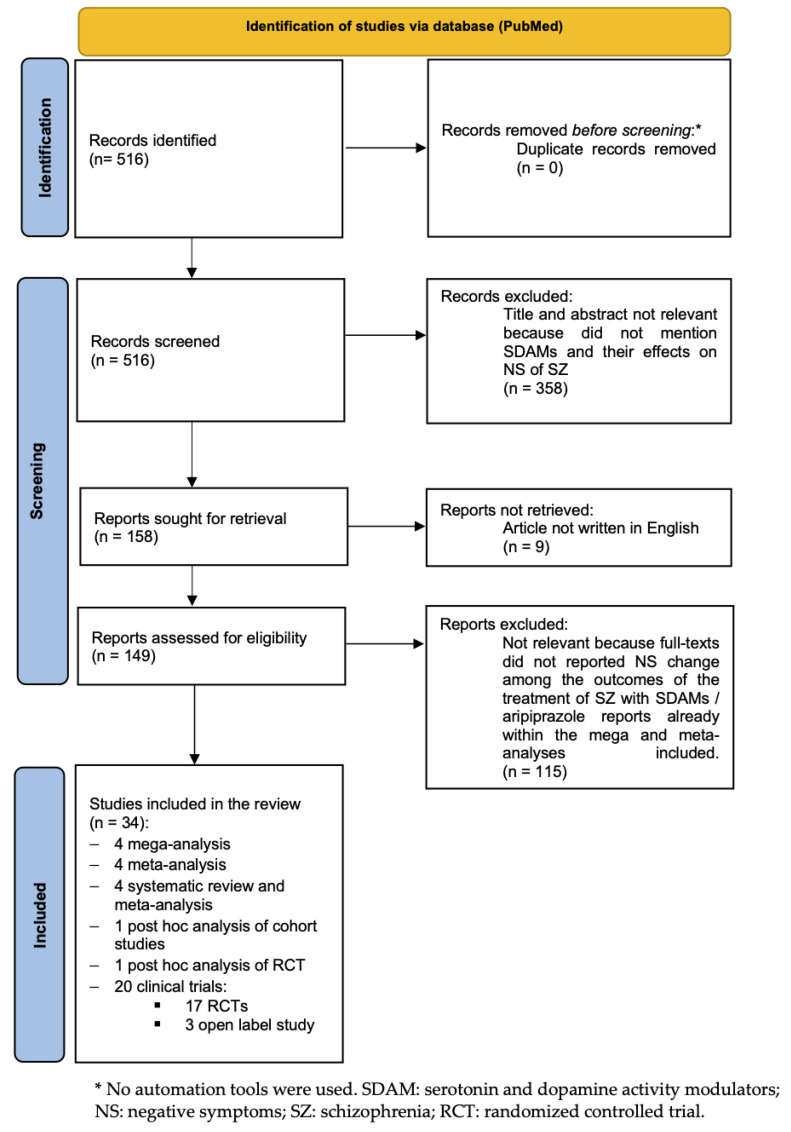
Selection flow-chart.

The screening and selection process is summarized in Figure 1.

### 2.4. Data Extraction

C.B. prepared a piloted form to define data to extract. G.C. extracted data, and R.S. checked for the correctness and completeness of extracted data. We limited extracted data using included systematic reviews and focusing exclusively on the PICOT of the research question [115].

### 2.5. Risk of Bias Assessment

Following the current guidelines on the methods to conduct rapid reviews [115,117,118,119], risk of bias was assessed in terms of study design and appropriateness of analyses related to the main outcome, i.e., efficacy of SDAMs on NS, by G.C. C.B. verified a 25% sample of study assessments. For the included systematic reviews and meta-analyses, the summary assessment of risk of bias proposed by the authors was accepted [115,117,118,119].

### 2.6. Synthesis and Discussion

A narrative knowledge synthesis in terms of a descriptive summary of the studies included was conducted by G.C. and R.S. [115,117,118,119]. C.B. discussed reasons for differences among studies in terms of heterogeneity of PICOT elements and study design, described potential limitations arising from methodological choices, and stated limitations with the conclusions of the current review based on limitations of the included literature and of the methods employed for this work [115,117,118,119]. P.R. supervised and verified the work of C.B. [115,117,118,119].

## 3. Results

Five hundred sixteen records were obtained from the search on PubMed. Following the algorithm described above and reported in Figure 1, 34 records were finally included in the review. In detail, four mega-analyses, four meta-analyses, four systematic reviews and meta-analyses, one post hoc analysis of cohort studies, one post hoc analysis of RCT, and 20 clinical trials (17 randomized controlled trials and three open-label studies) were included. The results are presented in four subsections, one for each of the four SDAMs analyzed. 

### 3.1. Studies on the Efficacy of Aripiprazole in the Treatment of NS

The studies about the efficacy of aripiprazole in the treatment of NS were grouped as follows: aripiprazole versus placebo, aripiprazole versus other antipsychotics, and aripiprazole as augmentation treatment.

#### 3.1.1. Aripiprazole versus Placebo

In this subsection, we considered two meta-analyses and six RCTs.

A systematic review and meta-analysis by Osugo et al. (2022) [126] evaluated the efficacy of dopamine partial agonists and of pro-dopaminergic drugs on NS of SZ. Aripiprazole was considered in thirteen studies, where it was compared to placebo. The change in PANSS negative subscales was considered among the primary outcomes. Even though this meta-analysis analyzed the change in NS in relation to the overall group of dopaminergic partial agonists, the authors reported an improvement in NS in the aripiprazole versus placebo pooled studies (standardized mean difference [SMD] −0.33; CI [−0.40, −0.26]) [126].

Fusar-Poli et al. (2015) [84] conducted a meta-analysis to evaluate the efficacy of a range of different pharmacological treatments (first- and second-generation antipsychotics, antidepressants, glutamatergic agents, combinations of pharmacological agents) versus placebo in patients diagnosed with SZ. NS change was considered among the primary outcomes and evaluated with the Positive and Negative Symptom Scale (PANSS) [127] negative subscale or with the SANS [54]. The meta-analysis included 168 original contributions. Three of them provided data on the effect of aripiprazole on NS. The overall estimate of the effectiveness of aripiprazole as compared to placebo was significant (SMD = −1.93; 95%; CI [−2.21, −1.66]; *p* value not available).

Kane et al. (2002) [128] compared aripiprazole (15 mg/d or 30 mg/d) versus placebo and haloperidol (10 mg/d) versus placebo in a 4-week double-blind, randomized study. This study was conducted at 36 U.S. centers, including a total amount of 414 adult patients diagnosed with SZ or schizoaffective disorder. NS change was evaluated among the primary outcomes with the PANSS negative subscale. At the endpoint, aripiprazole 15 mg/d showed a significantly greater improvement in the PANSS negative subscale compared to placebo (mean change –3.6; *p* = 0.006). A significant result was also produced by haloperidol versus placebo (mean change = –2.9; *p* = 0.043). An improvement in NS was observed in the aripiprazole 30 mg/d arm too; however, it was not significantly different from placebo. No direct comparison was conducted between haloperidol and aripiprazole, or between the two doses of aripiprazole [128].

Potkin et al. (2003) [129] conducted a 4-week randomized, controlled, double-blind study on 404 inpatients diagnosed with SZ or schizoaffective disorder, who were randomized to aripiprazole 20 mg/d, aripiprazole 30 mg/d, risperidone 6 mg/d, or placebo. From week 1 of observation to the endpoint (4 weeks), significantly greater improvements in the PANSS negative subscale (measured as secondary outcome) were seen for both doses of aripiprazole when compared to placebo (aripiprazole 20 mg, *p* = 0.002; aripiprazole 30 mg, *p* = 0.002). A similar result was observed for risperidone versus placebo. No direct comparison between aripiprazole and risperidone was conducted [129].

The efficacy of aripiprazole (10 mg/d, 15 mg/d, or 20 mg/d) versus placebo was also evaluated in a six-week randomized, double-blind, placebo-controlled study by McEvoy et al. including 420 patients diagnosed with SZ requiring hospitalization for an acute exacerbation. A significantly greater improvement in the PANSS negative subscale was observed among the secondary outcomes for all three doses of aripiprazole compared to placebo (*p* < 0.01) [130].

A multinational, randomized, double-blind study administered aripiprazole 10 mg/d for 6 weeks on a cohort of 152 patients with SZ compared to a placebo arm (*n* = 153). Patients were hospitalized for a week of washout from the previous antipsychotic and for a minimum of four of the total six weeks of treatment with aripiprazole. Subjects had at least 1 year of illness duration, acute symptomatology that required hospitalization, or antipsychotic change in the last year. NS change was a secondary outcome evaluated with the PANSS negative subscale and the 16-item Negative Symptom Assessment (NSA-16) [131]. As compared to placebo, significant differences in the PANSS negative subscale (least squares mean differences LSMD = −1.2; 95% CI [−2.2, −0.2], *p* < 0.05) and in the NSA-16 (LSMD [95% CI]: −4.2 [−6.4 to −2.0], *p* < 0.001) were observed [132].

Kane et al. (2014) [133] also conducted a randomized, double-blind, placebo-controlled study on 340 patients diagnosed with SZ that received treatment with 400 mg of aripiprazole once-monthly (AOM), a long-acting injectable suspension of aripiprazole. Changes in the PANSS negative subscale were evaluated every 2 weeks until the endpoint (10 weeks) as a secondary outcome. A significant difference between AOM and placebo was found throughout the whole period of observation: PANSS negative subscale was significantly improved from baseline at each time point (*p* < 0.0001) until the endpoint [133].

A multicenter, randomized, double-blind, placebo-controlled clinical trial compared 10 mg/d and 30 mg/d oral aripiprazole treatment with placebo, in a sample of 302 adolescents with SZ. Both 10 mg and 30 mg daily doses produced significantly greater results than placebo in improving the PANSS negative subscale. Only the 10 mg/d arm maintained significance at the end of the six-week study, while the 30 mg/d treatment was significant at weeks 3 and 4 (*p* < 0.05) but not at the study endpoint (*p* = 0.10) [134].

In conclusion, all of the studies cited showed a significant improvement in NS in patients treated with aripiprazole as compared to placebo.

#### 3.1.2. Aripiprazole versus Other Antipsychotics 

In this section, we analyzed one systematic review and network meta-analysis, one meta-analysis, and one post hoc analysis.

Huhn et al. (2019) [135] conducted a systematic review and network meta-analysis of placebo and head-to-head randomized controlled trials (RCTs) of 32 oral antipsychotics for the acute treatment of multi-episode schizophrenia, including 550 reports from 402 studies with 53,463 adult participants presenting an SZ spectrum disorder with acute symptoms. Change in NS was a secondary outcome measured with the PANSS negative subscale. One hundred and thirty-two studies (33% of the total studies included in the meta-analysis) evaluated NS. Within these RCTs, 1353 patients were treated with aripiprazole. As compared to placebo, aripiprazole showed greater efficacy in reducing NS (SMD = −0.33; 95% CI [−0.41, −0.24]). By means of a network analysis approach, the efficacy of aripiprazole versus placebo in reducing NS was indirectly compared to that of other antipsychotics compared to placebo. According to this specific type of analysis, aripiprazole showed greater efficacy than haloperidol, lurasidone, quetiapine, and cariprazine and less efficacy than ziprasidone, chlorpromazine, risperidone, olanzapine, amisulpride, and clozapine [135]. 

A meta-analysis including 150 double-blind studies (21,533 participants) compared second-generation versus first-generation antipsychotic (FGA) efficacy. The efficacy of the drugs in reducing NS was evaluated with the PANSS negative subscale. Data obtained from five double-blind studies (2049 patients) showed that the treatment with aripiprazole 10–30 mg/d was not superior to first-generation antipsychotics (*p* = 0.079) in terms of reduction of NS. As suggested by the authors, this negative result might depend on the specific meta-analytic approach [87].

Nielsen et al. (2022) [136] conducted a post hoc analysis of the data from two longitudinal cohort studies that administered a six-week treatment with amisulpride (*n* = 47) or aripiprazole (*n* = 48) to drug-naïve patients with SZ. The change in NS severity was the primary outcome of the study. NS were assessed with seven items of the PANSS as proposed by Wallwork et al. (2012) [137]. The two cohorts had similar baseline NS. A between-group difference in NS severity was found after 6 weeks of antipsychotic treatment (*p* = 0.037), with lower NS in the aripiprazole-treated cohort. A time effect in terms of reduction of NS severity was found in the aripiprazole treated group (*p* < 0.001) but not in the cohort treated with amisulpride (*p* = 0.23) [136].

The studies comparing the efficacy of aripiprazole on NS with those of other antipsychotics do not completely agree. Some authors found a superiority of aripiprazole in the treatment of NS as compared to haloperidol, lurasidone, quetiapine, cariprazine [135] and amisulpride [136], while Leucht et al. [87] did not find aripiprazole to be superior to FGA.

#### 3.1.3. Aripiprazole as Augmentation Treatment

We included two systematic reviews and meta-analyses, one meta-analysis, one RCT, and one open-label study that analyzed the efficacy of aripiprazole on NS when employed as augmentation of other antipsychotics. 

Galling et al. (2017) [138] conducted a systematic review, meta-analysis, and meta-regression analysis that included 31 studies of patients with SZ or schizoaffective disorder who received antipsychotic augmentation therapies. The study compared augmentation with different antipsychotics, with placebo, and continuation of previous antipsychotic monotherapy. NS variation was a secondary outcome assessed with the PANSS negative subscales and with the SANS. NS improvement was significant only in the eight studies where aripiprazole was chosen as augmentation therapy (*n* = 532, SMD = −0.41; 95% CI [−0.79, −0.03]; *p* = 0.036). A similar meta-analysis was produced by Zheng et al. (2016) [139] who considered 55 RCTs (4.457 patients) evaluating aripiprazole as augmentation therapy in SZ. Patients were randomized to augmentation with aripiprazole, augmentation with placebo, or continuation of previous antipsychotic treatment. NS change was a secondary outcome evaluated with the PANSS negative subscale. This study showed a significantly better efficacy of aripiprazole augmentation therapy in improving NS when compared to placebo augmentation or continuation of antipsychotic monotherapy (SMD = −0.61; 95% CI [−0.91, −0.31]; *p* < 0.00001).

Lee et al. [140] conducted a double-blind placebo-controlled study evaluating risperidone augmentation with aripiprazole (10 mg/d for 12 weeks) versus risperidone augmentation with placebo in patients diagnosed with SZ. The change in NS assessed with the PANSS negative subscale was included among the primary outcomes. After 12 weeks of evaluation, a second phase of the study was conducted. In the active arm, aripiprazole was titrated to a flexible dose, while risperidone was tapered in parallel. The placebo arm received risperidone only. A significantly greater improvement in NS was observed in the aripiprazole-treated arm, both at phase 1 and phase 2 endpoints, indicating a positive effect of aripiprazole on NS [140].

A systematic review and meta-analysis considered a total amount of 25 augmentation strategies (including antipsychotics, antidepressants, mood stabilizers, and other agents) for clozapine refractory SZ. Seven placebo-controlled trials considered clozapine augmentation with aripiprazole (for a total of 486 patients). NS change was evaluated among the secondary outcomes with the PANSS negative subscale and, where available, with the SANS. Results showed that augmentation with aripiprazole produced improvement in NS in five of the seven RCTs (*n* = 328; SMD = −0.33; 95% CI [−0.55, −0.11]; *p* < 0.05) [141]. Mitsonis et al. (2006) [83] also investigated the efficacy of clozapine augmentation with aripiprazole in an open-label study in which oral aripiprazole (15 mg/d) was administered to 27 stabilized adult outpatients diagnosed with SZ who were already being treated with clozapine (100–900 mg/d). A significant improvement in the mean scores for the PANSS negative subscale was observed at the endpoint, i.e., after 16 weeks of treatment (*p* < 0.001) [142].

According to all of the studies cited in this section, as compared to the placebo augmentation arm, the addition of aripiprazole to another antipsychotic, including clozapine, showed greater improvements in NS.

The studies on the efficacy of aripiprazole in the treatment of NS are summarized in Table 2.

### 3.2. Studies on the Efficacy of Brexpiprazole in the Treatment of NS

We included one meta-analysis, one systematic review and meta-analysis, three mega-analyses, five RCTs, and one open-label study that analyzed the efficacy of brexpiprazole in the treatment of NS.

Sabe M. et al. (2021) performed a dose–response meta-analysis of forty RCTs that examined the effectiveness of antipsychotics for the acute exacerbation of schizophrenia [143]. In three of these studies, brexpiprazole was examined at doses between 0.25 and 4 mg/d [144]. The authors estimated that a dose of 2.1 mg/d was sufficient to obtain the 95% effective dose (ED95), namely the dose required to achieve a significant effect in the 95% of the selected population in improving the PANSS negative subscale. The dose–response curve was plateau-shaped, suggesting that higher doses were not further efficacious in treating NS but also meant that a lower dose of antipsychotics could be as effective as higher doses [143]. According to these findings, patients treated with high doses of antipsychotics to control positive symptoms should also benefit from effective treatment of NS. The primary outcome was the change in NS without comparison with a placebo group.

A systematic review and meta-analysis by Osugo et al. (2022) evaluated the efficacy of dopamine partial agonists and pro-dopaminergic drugs on NS of SZ [126]. Brexpiprazole was considered in six studies, where it was compared with placebo. The change in PANSS negative subscales was considered among the primary outcomes. Even though this meta-analysis analyzed the change in NS in relation to the overall group of dopaminergic partial agonists, the authors reported an improvement in NS in the brexpiprazole versus placebo pooled studies (standardized mean difference [SMD] −0.22; CI [−0.31, −0.14]) [126].

Three randomized placebo-controlled trials, namely Correll et al. (2015) [144], Ishigooka et al. (2018) [145], and Kane et al. (2015) [146], were included in a mega-analysis by Kishi T et al. [147]. These three RCTs compared the efficacy and safety of 4 and 2 mg/d brexpiprazole for acute schizophrenia compared to placebo. Improvements in the PANSS negative subscale scores were chosen as secondary outcomes. The authors found a significant improvement in PANSS negative subscale scores in the arms treated with brexpiprazole 2 mg/d or 4 mg/d compared with the placebo arm [147]. No significant differences were found between 2 and 4 mg/d doses [147].

Meade et al. (2020) [148], conducted a mega-analysis of three short-term studies (6 weeks), randomized, double-blind, placebo-controlled studies [144] and two long-term (52 weeks) open-label extension studies [149]. In the short-term studies, 1405 patients received placebo (*n* = 527) or brexpiprazole 2–4 mg/d (*n* = 878). Some of the subjects enrolled in the brexpiprazole arm during the short-term trials participated in the long-term studies (*n* = 412 patients). In both short- and long-term studies, patients were stratified into two sub-groups according to symptoms severity. More severely ill were subjects with a PANSS total score > 95 (*n* = 215), and less severely ill were those with a PANSS total score ≤ 95 (*n* = 192). In the mega-analysis of the short-term studies, brexpiprazole showed greater improvement than placebo in both sub-groups in both the PANSS Negative subscale and in the PANSS-FSNS [148]. In the mega-analysis of the long-term studies, there was no focus on changes in NS from the baseline. Moreover, one of the two long-term studies did not find a significant improvement in NS compared to placebo after 52 weeks of treatment [150].

Marder et al. (2021) [151] reviewed the three short-term studies described above [144,146,152], focusing mainly on the mean change in PANSS item scores from baseline to week 6. They found a significant improvement (*p* < 0.01) for the PANSS items emotional withdrawal (N2), poor rapport (N3), lack of spontaneity and flow of conversation (N6), and more significant remission (*p* < 0.001) in passive/apathetic social withdrawal (N4) and active social avoidance (G16) [151].

In conclusion, most of the studies found that brexpiprazole 2–4 mg/d was better than placebo in the treatment of NS [144,145,146,147,148], while Fleischhacker et al. [150] did not find a significant difference after 52 weeks. We did not find any study comparing brexpiprazole with other antipsychotic drugs.

The studies on the efficacy of brexpiprazole in the treatment of NS are summarized in Table 3.

### 3.3. Studies on the Efficacy of Cariprazine in the Treatment of NS

The studies about the efficacy of cariprazine in the treatment of NS were grouped as follows: cariprazine versus placebo and cariprazine in the treatment of predominant NS and versus other antipsychotics.

#### 3.3.1. Cariprazine versus Placebo

We found one systematic review and meta-analysis, one mega-analysis, and three RCTs.

The efficacy of cariprazine in reducing negative symptoms after 6-week treatment was investigated in three RCTs of patients with an acute exacerbation of SZ [132]. In these three studies, cariprazine was compared to placebo and administered at the dose of 1.5 mg/d, 4.5 mg/d [153], 3 mg/d [153] or 6 mg/d [132]. Kane et al. used a dosage between 3 and 6 mg/d or between 6 and 9 mg/d [154]. Cariprazine treatment has been associated with a statistically significant improvement in the PANSS negative subscale score [153] and in the NSA-16 total score [153]. Cariprazine 3–6 mg/d dosage in the study of Kane et al. (2015) was not superior to placebo [154]. NS reduction was not the primary outcome of the studies.

Corponi et al. (2017) chose the three abovementioned RCTs [132,153,154] to carry out a mega-analysis of all of the subjects included in the three studies [155]. Patients were divided into a low-dose group (≤6 mg/d) and a high-dose group (≥6 mg/d). Authors found that Cariprazine was more effective than placebo in treating negative symptoms, both for high and low doses, with no difference between the two groups (low dose-group mean difference (MD) = 2.01; 95% CI [1.2–2.82]; high dose-group MD = 1.81, 95% CI [1.2–2.42].

A systematic review and meta-analysis by Osugo et al. (2022) evaluated the efficacy of dopamine partial agonists and pro-dopaminergic drugs on NS of SZ [126]. Cariprazine was considered in five studies where it was compared to placebo. The change in PANSS negative subscales was considered among the primary outcomes. Even though this meta-analysis analyzed the change in NS in relation to the overall group of dopaminergic partial agonists, the authors reported an improvement in NS in the cariprazine versus placebo pooled studies (standardized mean difference [SMD] −0.31; CI [−0.45, −0.17]) [126].

In conclusion, cariprazine seems to be more effective than placebo in the treatment of NS with a dosage between 1.5 mg/d and 6 mg/d.

#### 3.3.2. Cariprazine in the Treatment of Predominant NS and versus Other Antipsychotics

We included one RCT, one open-label study, and one post-hoc analysis.

In an open-label, non-controlled study, 60 patients with a confirmed ICD-10 diagnosis of SZ with predominant NS were enrolled [156]. NS were assessed with the PANSS. Predominant NS were defined with PANSS Marder’s Factor score for negative symptoms (PANSS-FSNS) ≥ 15 and PANSS-FS for positive symptoms (PANSS-FSPS) < 19. PANSS-FSNS includes the PANSS items blunted affect (N1), emotional withdrawal (N2), poor rapport (N3), passive/apathetic social withdrawal (N4), lack of spontaneity/flow of conversation (N6), motor retardation (G7), and active social avoidance (G16), while PANSS-FSPS consists of the PANSS items delusion (P1), hallucinatory behavior (P3), grandiosity (P5), and suspiciousness/persecution (P6). Patients recruited were treated with cariprazine for 4 weeks, with a starting daily dose of 1.5 mg followed by upward titration by 1.5 mg weekly up to 6 mg/d. Symptoms were assessed at baseline and at weeks 1, 2, and 4 with the PANSS and the Clinical Assessment Interview for Negative Symptoms (CAINS). Significant changes from baseline values for negative symptoms were found in CAINS and PANSS after 4-week treatment. More precisely, there was a significant improvement in two items of the PANSS negative subscale: emotional withdrawal (N2) and difficulty in abstract thinking (N5) [156].

Nemeth et al. (2017) [157] enrolled 461 patients with predominant NS of schizophrenia for at least 6 months before screening [157]. Predominant NS were defined as PANSS-FSNS ≥ 24 and a score ≥ 4 on at least two of the four negative symptoms among blunted affect, passive or apathetic social withdrawal, lack of spontaneity, and flow of conversation and low levels of positive symptoms, i.e., a PANSS-FSPS ≥ 19 and a score ≥ 4 on two or more positive PANSS items were exclusion criteria. Patients were randomized to receive 26 weeks of treatment with cariprazine at the target dosage of 4.5 mg/d (*n* = 227) or risperidone at the target dosage of 4 mg/d (*n* = 229). The primary endpoint was the change in PANSS-FSNS. Patients treated with cariprazine, as compared to the risperidone arm, demonstrated significantly greater improvements in the PANSS negative subscale score after week 14 [157].

Fleischhacker et al. (2019) [158] set up a post hoc analysis of the RCT described above [157] (*n* = 454 patients). The authors found that cariprazine 4.5 mg/d was significantly more effective than risperidone 4 mg/d in reducing the score of several items included in the PANSS negative subscale [158]. In particular, after 24 weeks, patients treated with cariprazine displayed better improvements in the PANSS items blunted affect (N1), emotional withdrawal (N2), poor rapport (N3), passive/apathetic social withdrawal (N4), and difficulty in abstract thinking (N5). No significant between-group difference was seen in the lack of spontaneity/flow of conversation (N6) and stereotyped thinking (N7) items, suggesting that risperidone may also be similarly effective in these domains [158].

The three studies summarized in this paragraph [156] excluded patients with moderate and severe depressive and extrapyramidal symptoms and verified that the improvement in negative symptoms was related to treatment, independently of depressive and extrapyramidal symptoms.

In conclusion, cariprazine was found to be effective [156] and better than risperidone [157,158] in the reduction of predominant NS.

The studies on the efficacy of cariprazine in the treatment of NS are summarized in Table 4.

### 3.4. Efficacy of Lumateperone in the Treatment of NS

We found and included the only two available RCTs that considered the effect of lumateperone on NS as compared to placebo.

Two RCTs analyzed the efficacy of lumateperone on NS after a 4-week treatment compared with placebo in patients with an acute exacerbation of SZ [159]. The acute exacerbation was defined as a BPRS score ≥ 40 and the onset of the acute episode within four weeks of screening. In Correll et al. (2020), 450 patients were randomized into three groups (lumateperone tosylate 40 mg/d, lumateperone tosylate 60 mg/d, and placebo) [159], while in Lieberman et al. (2016), 335 patients were randomized into four groups (lumateperone tosylate 120 mg/d, lumateperone tosylate 60 mg/d, risperidone 4 mg/d, and placebo) [160]. No significant results in improving PANSS negative subscale score compared to placebo were found for any dosage of lumateperone after 4 weeks of treatment [159]. The authors suggest that a monitoring duration of 4 weeks could be too short to assess the emergence of significant changes in NS.

Lumateperone seems not to be effective for NS in the first four weeks of treatment. At present, long-term studies are not available.

The studies on the efficacy of lumateperone in the treatment of NS are summarized in Table 5.

## 4. Discussion

Most of the studies summarized in the present review evidenced that, as compared to placebo, aripiprazole [84,87,135,136,161], cariprazine [132,154,161], and brexpiprazole [143,148,161] may reduce NS. Conversely, lumateperone seems not to improve NS [159,160]. Some evidence for the efficacy of aripiprazole in the treatment of NS was also found in treatment-resistant SZ, where this SDAM was employed as add-on therapy [138,139,141].

Focusing on the comparison with first- and second-generation antipsychotics, only cariprazine demonstrated evidence of greater efficacy than risperidone in the treatment of NS. Regarding aripiprazole, two studies found a better efficacy of this drug in reducing NS versus amisulpride [136] and risperidone [140]; however, both studies showed methodological weaknesses related to the evaluation of NS, such as not taking into account secondary NS or not using second-generation scales for the assessment of NS. Finally, two large meta-analyses demonstrated a non-superiority of aripiprazole in the treatment of NS as compared to other antipsychotics [87,135]. On this topic, we did not find any specific results for brexpiprazole and lumateperone.

Various methodological issues and the lack of studies on the role of SDAMs in the treatment of NS may lead to these fragmented results. In particular, we identified some concerns related to the study population, the experimental design, and the NS assessment. Study populations were heterogeneous within and between studies. Some included only patients with SZ [130,132,133,134,142,144,145,146,147,148,149,150,151,152,153,154,155,156,157,158,159,160], while others included both patients with SZ and patients with other diagnoses belonging to the SZ spectrum (DSM-5) [84,126,128,129,135,136,138,139,141]. One study focused on patients with first-episode psychosis [136]; all the others included patients with any duration of illness. One study analyzed only drug-naïve subjects [136], while others studied patients with treatment-resistant [138,139,140,141] and clozapine-refractory SZ [142]. Most of the studies evaluated the efficacy of the SDAMs during the acute phase of the disorder, i.e., with moderate-severe positive symptoms [128,129,130,132,133,144,145,146,147,148,150,151,152,153,154,155,159,160]. Only a few works included exclusively clinically stable patients with residual symptoms [142,149,150,156,158], and only three of them, testing the efficacy of cariprazine on NS, included exclusively patients with stable symptoms for at least six months [156,157,158]. This high variability within and between studies and the sometimes-small sample size of some experiments might reduce the chance of detecting a significant effect of SDAMs in the treatment of NS.

As for the design of the studies, we found a certain degree of heterogeneity. Most of the papers included in the present review were RCTs [128,134,140,144,145,146,149,150,152,153,154,157,158,159,160], while some were open-label studies [142,149,156]. Most RCTs compared SDAMs versus placebo, and only a few of them performed head-to-head comparisons between an SDAM and another class of antipsychotic [87,136,138,157,158]. Therefore, sound evidence on the efficacy of the SDAMs compared to other antipsychotics is still missing. In addition, the daily dose of the SDAMs was not homogenous between studies. Some studies did not establish fixed-dose treatments [87,136,140,148,149,151,152,154,155,158], others compared different daily doses [128,129,130,132,144,145,146,147,153,157,159,160], and only one work focused on the dose–effect relationship to treat NS [143]. These differences limit the comparison of the results between studies. Another crucial methodological problem concerned the choice of the primary outcome. Indeed, only 10 of the 34 included studies had the reduction of NS as a principal outcome [84,126,128,136,140,142,147,155,156,157]. In all other cases, studies were not designed to study NS, thus reducing the possibility of obtaining clear and solid information on the role of the SDAMs in the treatment of these symptoms. Eventually, the lack of real-world observational studies with a long-term follow-up could be another methodological factor behind the lack of robust scientific evidence on the usefulness of the SDAMs in the treatment of PNS. 

Finally, many methodological concerns were related to the assessment of NS. Only one study employed second-generation scales in addition to the first-generation ones [156]. This did not allow evaluation of the efficacy of SDAMs in reducing the subjective experience of NS. Moreover, many nonspecific items of first-generation scales were used, e.g., the PANSS items motor retardation (G6) and active social avoidance (G16), whose severity may depend on depressive and positive symptoms, respectively [144,145,146,148,149,150,151,157]. The evaluation of these nonspecific symptoms may increase the variability of data, thus reducing the possibility of observing a significant effect of the SDAMs treatment on NS. In particular, most of the studies included did not consider secondary NS. Only Ivanov et al. [156], Nemeth et al. [157], and Fleischhacker et al. [150] included patients with persistent predominant NS, excluding patients with moderate and severe depressive and extrapyramidal symptoms. The majority of the studies focused on the effect of the SDAMs in reducing all of the NS domains without any distinction between experiential and expressive factors or between the five domains. The only exceptions were Fleishhacker et al. (2019) [158] and Marder et al. (2021) [151], which focused on single items of the PANSS. Because of the methodological choice to pool together all of the NS domains, we could not understand if the SDAMs may be effective in the reduction of one or more specific NS domains. Moreover, if a drug had a selective efficacy in reducing one specific NS, this effect may have been “covered” by the other NS that did not respond to the pharmacological treatment. Finally, the assessment of NS was not sufficient in terms of duration or frequency [150,151,152,159,160]. Indeed, too-short follow-ups and infrequent evaluations of symptoms may not detect a time effect in NS reduction related to the treatment with SDAMs.

Another reason for the lack of evidence of the efficacy of the SDAMs in the treatment of NS was the relatively short period of time that had elapsed following their FDA approval. Possible ways to go beyond these limits are large umbrella trials evaluating different SDAMs and second-generation antipsychotics against a single placebo arm and large, long-term, real-world observational studies. The study samples may include patients clinically stabilized, without treatment-resistant SZ, with a homogenous duration of illness—e.g., maximum 5 years—and with PNS evaluated with first- and second-generation tools. The effect of antipsychotic treatment on the experiential and expressive factors or on each of the five domains of NS should be the primary outcome of the study. A regular and concomitant assessment of positive, depressive, and extrapyramidal symptoms is needed to control for potential secondary NS.

### 4.1. Limitations and Strengths

The principal limitations of this work are the rapid nature of the review, which did not allow for accurate screening of the methodological quality of the included records, and the choice to search exclusively the PubMed platform and no other databases such as Web of Science.

The main strength of this paper is the choice of the topic covered, previously not addressed by other reviews. Indeed, this rapid review represents the first synthesis of the evidence on the efficacy of the four SDAMs approved for the treatment of NS in SZ.

### 4.2. Implications and Future Directions

The main implications of the findings of this review concern the use of cariprazine and lumateperone to treat NS. In particular, cariprazine was the only SDAM that demonstrated, in large studies with sound methodology, a superiority to an SGA while lumateperone, contrary to the other three SDAMs, did not show superiority to placebo. For this drug, further studies with longer follow-ups are needed. Moreover, the current rapid review highlights the need for new, methodologically sound studies aimed at demonstrating the efficacy of SDAMs in the reduction of NS. This goal may be achieved by selecting samples of patients with SZ homogenous in terms of duration of illness, with a detailed characterization of persistent NS assessed with first- and second-generation scales and grouped in the experiential and expressive factors. Three study designs may be suitable for this purpose, namely long-term and significantly large sample RCTs to determine the duration of treatment and the maintenance of antipsychotic doses [162], head-to-head comparisons of SDAMs with other antipsychotics and between SDAMs themselves, and long-term, real-world observational studies. Another possibility is the study of new SDAMs. Among these should be mentioned allosteric modulators of both dopamine and serotonin receptors. Compared to the available SDAMs that directly compete with the physiological ligand, these compounds may exhibit an increased selectivity for G protein-coupled subunits and the potential to preserve activity dependence and both spatial and temporal characteristics of endogenous physiological ligands [163]. Further evidence is needed to fill the gap in knowledge about possible treatments for NS. This line of research is extremely important to reduce the burdens of these disabling symptoms that represent an important obstacle for patients’ and caregivers’ quality of life.

## 5. Conclusions

In conclusion, the SDAMs represent a possible option for the treatment of NS in SZ. However, the evidence on the efficacy of these drugs is still poor and fragmented because of the lack of studies on this topic and of several methodological issues concerning the choice of the sample, the evaluation of NS, the study design, the follow-up duration, and the comparators, mainly placebo and not another antipsychotic. Only a few studies, mainly on cariprazine in monotherapy, confirmed the superiority of this group of drugs to other classes of antipsychotics. Therefore, according to the available evidence synthesized in the current review, among SDAMs and antipsychotics in general, only cariprazine may represent an effective strategy to reduce these disabling symptoms. New, methodologically robust studies focusing on well-characterized patients with head-to-head comparisons of antipsychotic treatments and long-term real-world observational studies are needed to fill this knowledge gap.

## Figures and Tables

**Table 1:** Human receptor affinity of serotonin and dopamine activity modulators (SDAMs) approved * for the treatment of schizophrenia and potential clinical effect related to the drug–receptor interaction.

Receptor	Type of Activity	Affinity Ki (Nm) In Vitro			Potential Clinical Effect Related to the Drug–Receptor Interaction [101,107]	
		Aripiprazole [101]	Cariprazine [99,101]	Brexpiprazole [101,104,105]	Lumateperone [108,113,114]	
Dopamine D1	Antagonist	387	N/A	164	52 **	Antipsychotic effect, sedation, may contribute to reduce stereotypies
Dopamine D2	Partial Agonist	1.4	0.59	0.3	32 ***	Antipsychotic effect, EPS ****, prolactin elevation, akathisia, nausea, insomnia, subjective response to treatment
	*Intrinsic activity*	60%	30%	45%	N/A ***	
Dopamine D3	Partial Agonist	1	0.085	1.1	N/A	Effects on positive and negative symptoms, procognitive effect, EPS, akathisia.
	Intrinsic activity	28%	71%	15%		
Dopamine D4	Antagonist	216.5	N/A	6.3	<100	Antidepressant and anxiolytic effects, procognitive effect, reduction of EPS
Serotonin 5-HT1A	Partial Agonist	5.6	3	0.12	N/A	Anxiolytic effect, may contribute to boost antidepressant action
	Intrinsic activity	73%	39%	60%	N/A	
Serotonin 5-HT2A	Antagonist	8.7	19	0.47	0.54	Reduction of EPS, weight gain
Serotonin 5-HT2B	Antagonist	0.36	0.58	1.9	>1000	Not known
Serotonin 5-HT2C	Antagonist	18.7	134	34	173	Weight gain

* Approved by the FDA as of 31 December 2022. ** Lumateperone indirectly modulates glutamatergic neurotransmission by activating dopamine D1 receptors that increase the phosphorylation of GluN2B-type N-methyl-d-aspartate (NMDA) receptors in mesolimbic/mesocortical dopamine systems [108]. This action increases the number of NMDA channels in the membrane of prefrontal neurons, enhancing glutamatergic signaling [112]. *** In vitro studies found that lumateperone acts as an antagonist to postsynaptic D2 receptors [108], but it is still unclear if it acts as an agonist or an antagonist on presynaptic D2 receptors [109]. **** EPS: extra-pyramidal symptoms.

**Table 2:** Studies on the efficacy of aripiprazole in the treatment of negative symptoms.

Study	Study Design	Drugs	Comparator Group(s)	Sample	Age	Sample Characteristics	Diagnosis	Assessment of NS	Outcome	Results	
										Change in NS	Effect
Osugo et al., 2022 [126]	Systematic review and meta-analysis	ARI	placebo	Studies N = 13 patients *n* = 4.960	N/A	RCTs of dopamine partial agonist monotherapy vs. placebo.	SZ, SZA	PANSS negative	Primary outcome: change in PANSS total, positive and negative	↑ vs. placebo	PANSS negative SMD= −0.33; CI 95% [−0.40, −0.26]
Fusar-Poli et al., 2015 [84]	Meta-analysis	ARI	placebo	Studies N = 3 patients *n* = 751	≥18	Original articles on NS treatment in adults.	SZ, SZA	PANSS negative, SANS	Primary outcome: change in PANSS negative, change in SANS	↑ vs. placebo	PANSS negative SMD = −1.93; CI 95% [−2.21, −1.66]
Kane et al., 2002 [128]	Double blind RCT Multicentric (36 US medical centers)	ARI 15 mg/d; ARI 30 mg/d	placebo	Patients *n* = 414	ARI 15 mg/d: 37.8 (1.0); ARI 30 mg/d: 39.3 (1.0); Placebo: 38.5 (0.9)	Patients with acute relapse.	SZ (71% of placebo, 73% of ARI 15 mg/d, 71% of ARI 30 mg/d), SZA	PANSS negative	Primary outcome: change in PANSS negative	↑ ARI 15 mg/d vs. placebo	PANSS negative mean change –3.6; *p* = 0.006
Potkin et al., 2003 [129]	Double blind RCT multicentric (40 US medical centers)	ARI 20 mg/d; ARI 30 mg/d	placebo	404	ARI 20 mg/d: 30.1; ARI 30 mg/d: 40.2; Placebo: 38.8	Patients with acute relapse.	SZ (72%), SZA (28%)	PANSS negative	Primary outcome: change in PANSS total and change in PANSS positive; secondary outcome: change in PANSS negative	↑ vs. placebo	PANSS negative significant improvement vs. placebo (ARI 20 mg/d, *p* = 0.002; ARI 30 mg/d, *p* = 0.002)
McEvoy et al., 2007 [130]	Double blind RCT	ARI 10 mg/d; ARI 15 mg/d; ARI 20 mg/d	placebo	420	ARI 10 mg/d: 40.0 (1.1); ARI 15 mg/d: 40.0 (1.1); ARI 20 mg/d: 40.4 (1.1); Placebo: 41.2 (1.1)	Patients with acute relapse requiring hospitalization.	SZ	PANSS negative	Primary outcome: change in PANSS total; secondary outcome: PANSS negative	↑ vs. placebo	PANSS negative improvement in all ARI arms was greater than placebo (*p* ≤ 0.01)
Durgam et al., 2015 [132]	Double blind RCT, phase III	ARI 10	placebo	617	ARI 10 mg/d: 39.3 (10.8); placebo: 38.2 (11.3)	Patients with acute relapse requiring hospitalization.	SZ	PANSS negative, NSA-16	Primary outcome: change in PANSS total; secondary outcome: change in PANSS negative, NSA-16	↑ vs. placebo	PANSS negative mean difference: −1.2, *p* < 0.05; NSA-16 mean difference: −4.2, *p* < 0.001
Kane et al., 2014 [133]	Double blind RCT, phase III, multicentric (41 centers in US, Croatia, Latvia)	ARI once-monthly (AOM) 400 mg	placebo	340	AOM 400 mg: 42.1 (11.0); Placebo: 42.7 (10.9)	Patients with acute relapse requiring hospitalization.	SZ	PANSS negative	Primary outcome: change in PANSS total; secondary outcome: change in PANSS negative	↑ vs. placebo	LSMC in PANSS negative for ARI was significantly improved vs. placebo, *p* < 0.0001
Findling et al., 2008 [134]	Double blind RCT, phase III, multicentric (101 centers in US, EU, South America, Asia, Caribbean, South Africa)	ARI 10 mg/d; ARI 30 mg/d	placebo	302	ARI 10 mg/d: 15.6 (1.3); ARI 30 mg/d: 15.4 (1.4); Placebo: 15.4 (1.4)	Adolescent patients (13 to 17 years).	SZ	PANSS negative	Primary outcome: change in PANSS total; secondary outcome: change in PANSS negative	↑ ARI 10 mg/d vs. placebo	PANSS negative improvement in ARI 10 vs. placebo *p* = 0.05
Huhn et al., 2019 [135]	Systematic review and network meta-analysis	32 oral antipsychotics included ARI	placebo	Studies N= 402; patients *n* = 53,463	37.40 (5.96)	Of the studies included: articles comparing oral SGA vs. placebo / oral FGA vs. placebo.	SZ, SFD, SZA	PANSS negative	Primary outcome: change in PANSS total; secondary outcome: change in PANSS negative	↑ vs. placebo	PANSS negative SMD = −0.33; 95% CI [−0.41, −0.24].
Leucht et al., 2009 [87]	Meta-analysis	SGA, included ARI 10–30 mg/d	FGA	Studies N= 5; Patients *n* = 2049	36.2 (7.1)	Original articles comparing oral SGA with FGA for treatment of SZ, SFD, SZA, DD	SZ, SFD, SZA, DD	PANSS negative	Primary outcome: change in PANSS total; secondary outcome: change in PANSS negative	↔ vs. FGA	PANSS negative change was not significantly different from first-generation antipsychotic drug (*p* = 0.079)
Nielsen et al., 2022 [136]	Post hoc analysis of two longitudinal cohort studies	ARI 10 mg/d ± 4.7, 2.5–25 (range)	AMI 276 mg/d ± 173, 50–800 (range)	95	ARI 22.9 (4), 18–42 (range); AMI 24.5 (6), 18–43 (range)	Patients from psychiatric hospitals and outpatient clinics, antipsychotic naïve.	SZ (71% ARI, 96% AMI), SZA (2% ARI, 4% AMI), non-affective psychoses other than SZ (27% ARI)	PANSS 7-items NS dimension according to Wallwork [137]	Primary Outcome: change in PANSS 7-items NS dimension according to Wallwork [137]	↑ vs. AMI	Between-group difference in NS severity (*p* = 0.037) with lower NS in the ARI treated cohort
Galling et al., 2017 [138]	Systematic review and meta-analysis	CLO + FGA; CLO + SGA; FGA + SGA; SGA + SGA	augmentation with placebo or continuation of antipsychotic monotherapy	Studies N = 31; patients *n* = 4136	N/A	Of the studies included: RCTs with ≥20 adults on antipsychotic augmentation vs. placebo augmentation or single antipsychotic continuation	SZ, SZA	PANSS negative, SANS	Primary outcome: change in PANSS total; secondary outcome: change in PANSS negative, SANS	↑ with ARI augmentation	PANSS negative SMD= −0.41; 95% CI [−0.79, −0.03]; *p* = 0.036
Zheng et al., 2016 [139]	Meta-analysis	augmentation with ARI 14.0 mg/d (mean) ± 7	augmentation with placebo or antipsychotic monotherapy	Studies N = 55; patients *n* = 4457	34.9 (6.0)	Of the studies included: original studies on ARI augmentation. Of the patients included: illness duration 7.0 ± 6.3 years	SZ (98%), SZA (2%)	PANSS negative	Primary outcome: change in PANSS total; secondary outcome: change in PANSS negative	↑ vs. placebo or antipsychotic monotherapy	PANSS negative SMD= −0.61; 95% CI [−0.91, −0.31]; *p* < 0.00001
Lee et al., 2013 [140]	Double blind RCT, multicentric (3 hospitals in Korea)	RIS (3–6 mg/d) augmentation with ARI 10 mg/d	RIS (3–6 mg/d) augmentation with placebo	35	ARI 10 mg/d: 51.00 (2.32); Placebo: 50.50 (2.87)	Inpatients stabilized with RIS (3–6 mg/d) for 3 months.	SZ	PANSS negative	Primary outcome: change in PANSS negative	↑ vs. placebo	PANSS negative significant difference between groups in both phases of the study (*p* < 0.05)
Siskind et al., 2018 [141]	Systematic review and meta-analysis (Studies from China, Italy, Korea)	CLO augmentation with ARI	CLO augmentation with placebo	Studies N= 7; patients *n* = 486	N/A	Of the studies included: original studies comparing CLO plus ARI vs. CLO plus placebo.	SZ, SZA	PANSS negative, SANS	Primary outcome: change in PANSS total; secondary outcome: change in PANSS negative	↑ vs. placebo augmentation in 5 studies	PANSS negative SMD = −0.33, 95% CI [−0.55, −0.11]; *p* < 0.05
Mitsonis et al., 2006 [142]	Open-label pilot study	CLO augmentation with ARI 15 mg/d	CLO augmentation with placebo	27	41.9 (8.6)	Stable outpatients with residual symptoms after CLO treatment ≥ 1 year.	SZ	PANSS negative	Primary outcome: change in PANSS negative	↑ vs. placebo	PANSS negative mean scores improved vs. placebo *p* < 0.001

↑ better results; ↔ similar results; AMI: amisulpride; AOM: aripiprazole once monthly; ARI: aripiprazole; CI: confidence interval; CLO: clozapine; DD: delusional disorder; EU: Europe; FGA: first-generation antipsychotic; LSMC: least square mean change; N: number of studies; *n* = number of patients; N/A: not applicable; NS: negative symptoms; NSA-16: 16-item Negative Symptom Assessment; PANSS: Positive and Negative Symptoms Scale; RCT: randomized control trial; RIS: risperidone; SANS: Scale for the Assessment of Negative Symptoms; SD: standard deviation; SFD: schizophreniform disorder; SMD: standardized mean difference; SZ: schizophrenia; SZA: schizoaffective disorder; US: United States; vs.: versus.

**Table 3:** Studies on the efficacy of brexpiprazole in the treatment of negative symptoms.

Study	Study Design	Drugs	Comparator Group(s)	Sample	Age	Sample Characteristics	Diagnosis	Assessment of NS	Outcome	Results	
										Change in NS	Effect
Sabe et al., 2021 [143]	Meta-analysis	BRE 0.25–4 mg/d	N/A	Studies N = 3 patients *n* = 1756	18–65 years (mean value and SD not available)	Patients with acute relapse.	SZ	PANSS negative subscale total score	Determination of the ED95 for NS	N/A	BRE 2.1 mg/d was sufficient to obtain the 95% effective dose (ED95)
Correll et al., 2015 [144]	Double blinded RCT, phase III	BRE 0.25 mg/d BRE 2 mg/d BRE 4 mg/d	placebo	623	BRE 0.25: 40.5 (11.4) BRE 2 mg/d: 39.6 (10.2) BRE 4 mg/d: 40.8 (11.0) placebo: 39.7 (10.8)	Patients with acute relapse. Excluded first psychotic episode Ethnicity: African American, Asian, White, Other	SZ	PANSS negative subscale total score, PANSS MF Negative Total Score	Primary outcome: change in PANSS total score Secondary outcome: change in PANSS negative, PANSS MF Negative score	↑ vs. placebo for BRE 2 mg/d and 4 mg/d	BRE 0.25 mg/d vs. placebo: PANSS Negative MD −1.07, *p* = 0.1; PANSS MF Negative Score MD −0.86, *p* = 0.20; BRE 2 mg/d vs. placebo: PANSS Negative MD −1.78, *p* = 0.0007; PANSS MF Negative Score MD −1.68, *p* = 0.002; BRE 4 mg/d vs. placebo: PANSS Negative MD −1.41, *p* = 0.007; PANSS MF Negative Score MD −1.30, *p* = 0.02.
Ishigooka et al., 2018 [145]	Double blinded RCT, phase III	BRE 1 mg/d, 2 mg/d, 4 mg/d	placebo	459	BRE 1 mg/d: 44.7 (11.5) BRE 2 mg/d: 43.3 (12.0) BRE 4 mg/d: 44.1 (11.9) placebo: 45.0 (11.3)	Patients with acute relapse. Excluded first psychotic episode. Ethnicity not available.	SZ	PANSS negative subscale total score, PANSS MF Negative Total Score	Primary outcome: change in PANSS total score Secondary outcome: change in PANSS negative, PANSS MF Negative score	↑ vs. placebo for BRE 2 mg/d and 4 mg/d	BRE 1 mg/d vs. placebo: PANSS Negative MD −1.14, *p* = 0.14; PANSS MF Negative Score MD −1.82, *p* = 0.02; BRE 2 mg/d vs. placebo: PANSS Negative MD −2.28, *p* = 0.002; PANSS MF Negative Score MD −2.57, *p* = 0.001; BRE 4 mg/d vs. placebo: PANSS Negative MD −2.04, *p* = 0.008; PANSS MF Negative Score MD −2.54, *p* = 0.001.
Kane et al., 2015a [146]	Double blinded RCT, phase III	BRE 1 mg/d, 2 mg/d, 4 mg/d	placebo	674	BRE 1 mg/d: 39.1 (11.9) BRE 2 mg/d: 38.6 (11.0) BRE 4 mg/d: 36.9 (10.9) placebo: 39.3 (10.8)	Patients with acute relapse. Excluded first psychotic episode. Ethnicity: African American, Asian, White, American Indian/Alaska Native, Other	SZ	PANSS negative subscale total score, PANSS MF Negative Total Score	Primary outcome: change in PANSS total score Secondary outcome: change in PANSS negative, PANSS MF Negative score	↑ vs. placebo only for BRE 4 mg/d	BRE 1 mg/d vs. placebo: PANSS Negative LSMD −0.78, *p* = 0.2; PANSS MF Negative Score LSMD −1.00, *p* = 0.10; BRE 2 mg/d vs. placebo: PANSS Negative LSMD −0.77, *p* = 0.15; PANSS MF Negative Score LSMD −0.98, *p* = 0.07; BRE 4 mg/d vs. placebo: PANSS Negative LSMD −1.22, *p* = 0.02; PANSS MF Negative Score LSMD −1.28, *p* = 0.01.
Osugo et al., 2022 [126]	Systematic review and meta-analysis	BRE	placebo	Studies N= 6 patients *n* = 2690	N/A	RCTs of dopamine partial agonist monotherapy vs. placebo.	SZ, SZA	PANSS negative	Primary outcome: change in PANSS total, positive and negative	↑ vs. placebo	PANSS negative SMD = −0.22; CI [−0.31, −0.14]
Kishi et al., 2018 [147]	Mega-analysis	BRE 2 mg/d BRE 4 mg/d	placebo	1444	18–65 years (mean value and SD not available)	Patients with acute relapse. Excluded first psychotic episode. Ethnicity: African American, Asian, White, Other	SZ	PANSS negative total score	Primary outcome: change in PANSS total, positive and negative subscale.	↑ vs. placebo BRE 2 mg/d ↔ BRE 4 mg/d	BRE 2 mg/d vs. placebo: PANSS negative SMD −0.32, *p* = 0.001; BRE 4 mg/d vs. placebo: PANSS negative SMD −0.30 *p* < 0.00001; BRE 2 mg/d vs. BRE 4 mg/d: PANSS negative SMD −0.01 *p* = 0.9.
Meade et al., 2020 [148]	Mega-analysis	Short-term studies: BRE 2–4 mg/d Long-term studies: BRE 1–4 mg/d	placebo	1405	short-term studies: BRE 2–4 mg/d msi: 38.6 (10.9) placebo msi: 38.9 (10.8) BRE 2–4 mg/d lsi: 39.6 (10.8) placebo lsi: 41.0 (10.5) long-term studies: BRE 1–4 mg/d msi: 38.1 (10.5) BRE 1–4 lsi: 39.3 (10.6)	Patients with acute relapse. Excluded first psychotic episode. Ethnicity: African American, Asian, White, Other	SZ	PANSS negative subscale total score, PANSS MF Negative Total Score	Primary outcome: change in PANSS total score Secondary outcome: change in PANSS negative, PANSS MF Negative score for short term studies.	↑ vs. placebo only for short-term studies.	BRE 2–4 mg/d msi vs. placebo: PANSS Negative LSMD −2.00, *p* = 0.0001; PANSS MF Negative Score LSMD −1.28, *p* = 0.001; BRE 2–4 mg/d lsi vs. placebo: PANSS Negative LSMD −0.88, *p* = 0.012; PANSS MF Negative Score LSMD −0.96, *p* = 0.001.
Forbes et al., 2018 [149]	Open- label phase III	BRE 1–4 mg/d	N/A	1072	40.0 (11.1)	Patients with a stable state with antipsychotic regimen for at least one 3-month period in the last year.	SZ	PANSS negative subscale total score, PANSS MF Negative Total Score	Primary outcome: frequency of AEs	N/A	Mean change in PANSS negative from baseline to week 52 was −2.8 (4.6); Mean change in PANSS MF Negative from baseline to week 52 was −2.8 (4.4)
Fleischhacker et al., 2017 [150]	Double blinded RCT, phase III	BRE 1–4 mg/d	placebo	524	BRE 38.8 (10.7) placebo 41.6 (10.6)	Patients with acute relapse. Excluded first psychotic episode.	SZ	PANSS negative subscale total score, PANSS MF Negative Total Score	Primary outcome: change in PANSS total score Secondary outcome: change in PANSS negative, PANSS MF Negative score	↔ vs. placebo	BRE 1–4 mg/d vs. placebo: PANSS negative LSMD −1.24, *p* = 0.05; PANSS MF Negative LSMD −1.23, *p* = 0.06
Marder et al., 2021 [151]	Mega analysis	short-term studies: BRE 2–4 mg/d long-term studies: BRE 1–4 mg/d	placebo	1778	short-term studies: BRE 2–4 mg/d: 39.1 (10.9) placebo: 39.8 (10.8) long-term studies: BRE 1–4 mg/d: 38.8 (10.7) placebo: 41.6 (10.6)	Patients with acute relapse. Excluded first psychotic episode. Ethnicity: white, other.	SZ	PANSS negative subscale total score, PANSS MF Negative Total Score	Primary outcome: change in PANSS total score Secondary outcome: changes in PANSS MF Negative score and in single items of PANSS MF;	↑ vs. placebo for both short-term and long-term studies	BRE 2–4 mg/d short-term studies single-item negative symptoms improvement vs. RIS 4 mg/d: N2,N3,N6 *p* < 0.01 N4, G16 *p* < 0.001; BRE 1–4 mg/d long-term studies vs. placebo: PANSS MF Negative LSMD −1.23, *p* = 0.063

↑ better results; ↔ similar results; BRE: brexpiprazole; CI: confidence interval; LSMD: least square mean difference; MD: mean difference; N: number of studies; n: number of patients; N/A: not applicable; NS: negative symptoms; PANSS: Positive and Negative Symptoms Scale; PANSS MF: Positive and Negative Symptoms Scale Marder Factor; QUE: quetiapine; QUE XR: quetiapine extended release; RCT: randomized control trial; RIS: risperidone; SMD: standardized mean difference; SZ: schizophrenia; vs.: versus; msi: more severely ill; lsi: less severely ill.

**Table 4:** Studies on the efficacy of cariprazine in the treatment of negative symptoms.

Study	Study Design	Drugs	Comparator Group(s)	Sample	Age	Sample Characteristics	Diagnosis	Assessment of NS	Outcome	Results	
										Change in NS	Effect
Durgam et al., 2014 [153]	Double blinded RCT, phase II	CAR 1.5 mg/d, 3 mg/d,4.5 mg/d	RIS 4 mg/d, placebo	732	CAR 1.5 mg/d: 36.8 (9.6) CAR 3 mg/d: 37.1 (10.4) CAR 4.5 mg/d: 35.8 (10.8) RIS 4 mg/d: 36.5 (11.1) placebo: 36.0 (10.8)	Patients with current exacerbation less than 2 weeks duration. Ethnicity: African American, Asian, White, Other	SZ	PANSS negative subscale total score, NSA-16 total score	Primary outcome: change in PANSS total score Secondary outcome: change in PANSS negative subscale and in NSA-16 total score	↑ vs. placebo	CAR 1.5 mg/d vs. placebo: PANSS negative LSMD −2.2, *p* < 0.001; NSA-16 LSMD −2.2, *p* < 0.001; CAR 3 mg/d vs. placebo: PANSS negative LSMD −2.5, *p* < 0.001; NSA-16 MD −4.6, *p* < 0.001 CAR 4.5 mg/d vs. placebo: PANSS negative LSMD −3.0, *p* < 0.001; NSA-16 MD −5.5, *p* < 0.001 RIS vs. placebo: PANSS negative LSMD −3.1, *p* < 0.001; NSA-16 MD −5.9, *p* < 0.001
Durgam et al., 2015 [132]	Double blinded RCT, phase III	CAR 3 mg/d, CAR 6 mg/d	ARI 10 mg/d, placebo	617	CAR 3 mg/d: 37.9 (10.6) CAR 6 mg/d: 38.6 (10.6) ARI 10 mg/d: 39.3 (10.8) placebo: 38.2 (11.3)	Patients with current exacerbation less than 2 weeks duration. Ethnicity: African American, White, Other	SZ	PANSS negative subscale total score, NSA-16	Primary outcome: change in PANSS total score Secondary outcome: change in PANSS negative, NSA-16	↑ vs. placebo ↔ vs. ARI	CAR 3 mg/d vs. placebo: PANSS negative LSMD −1.4, *p* < 0.01; NSA-16 LSMD −3.6, *p* < 0.01. CAR 6 mg/d vs. placebo: PANSS negative LSMD −1.7, *p* < 0.001; NSA-16 LSMD −4.5, *p* < 0.001; ARI 10 mg/d/day vs. placebo: PANSS negative LSMD: −1.2, *p* < 0.05; NSA-16 LSMD −4.2, *p* < 0.001
Corponi et al., 2017 [155]	Mega-analysis	CAR 1.5–4.5 mg/d CAR 6–9 mg/d	placebo	1795	18–65 (mean value and SD not available)	Patients with current exacerbation less than 2 weeks duration.	SZ	PANSS negative subscale total score	Change in PANSS total score and PANSS-subscales score	↑ vs. placebo	CAR 1.5–4.5 mg/d MD = 2.01; 95% Confidence Interval (CI): 1.2–2.82; CAR 6–9 mg/d MD = 1.81, 95% CI: 1.2–2.4
Osugo et al., 2022 [126]	Systematic review and meta-analysis	CAR	placebo	Studies N= 5 patients *n* = 2365	N/A	RCTs of dopamine partial agonist monotherapy vs. placebo.	SZ, SZA	PANSS negative	Primary outcome: change in PANSS total, positive and negative	↑ vs. placebo	PANSS negative SMD = −0.31; CI [−0.45, −0.17]
Ivanov et al., 2022 [156]	Open label, non-controlled study	CAR 1.5 mg/d–6 mg/d	N/A	60	35.6 (9.1)	Patients in stable state for ≥6 months. Presence of PPNS and low levels of positive symptoms.	SZ	PANSS negative subscale total score, CAINS total score	Primary outcome: change in PANSS negative and CAINS	↑ vs. baseline	CAR 1.5 mg/d–6 mg/d vs. baseline: PANSS negative MD −4.3, *p* < 0.01; CAINS MD −4.9, *p* < 0.01
Nemeth et al., 2017 [157]	Double blinded RCT, phase III	CAR 3 mg/d, 4.5 mg/d or 6 mg/d	RIS 3 mg/d, 4 mg/d or 6 mg/d	461	CAR group: 40.2 (10.5) RIS group: 40.7 (11.2)	Patients in stable state for ≥6 months. Presence of PPNS and low levels of positive symptoms. Ethnicity: White	SZ	PANSS negative subscale total score	Change in PANSS MF Negative score	↑ vs. RIS	CAR vs. RIS: LSMD −1.46, *p* = 0.002
Fleischhacker et al., 2019 [158]	Post hoc analysis of one RCT	CAR 4.5 mg/d	RIS 4 mg/d	454	18–65 years (mean value and SD not available)	Patients in stable state for ≥6 months. Presence of PPNS and low levels of positive symptoms.	SZ	PANSS negative subscale total score	Primary outcome: changes from baseline in individual items of PANSS	↑ vs. RIS	CAR 4.5 mg/d single-item negative symptoms improvement vs. RIS 4 mg/d: N1, N2, N3 *p* < 0.01 N4, N5 *p* < 0.05

↑ better results; ↔ similar results; CAINS: Clinical Assessment Interview for Negative Symptoms; CAR: cariprazine; CI: confidence interval; LSMD: least square mean difference; MD: mean difference; N: number of studies; n: number of patients; N/A: not applicable; NS: negative symptoms; NSA-16: 16-item Negative Symptom Assessment; PANSS: Positive and Negative Symptoms Scale; PANSS MF: Positive and Negative Symptoms Scale Marder Factor; PPNS: persistent predominant negative symptoms; RCT: randomized control trial; RIS: risperidone; SMD: standardized mean difference; SZ: schizophrenia; SZA: schizoaffective disorder; vs.: versus.

**Table 5:** Studies on the efficacy of lumateperone in the treatment of negative symptoms.

Study	Study Design	Drugs	Comparator Group(s)	Sample	Age	Sample Characteristics	Diagnosis	Assessment of NS	Outcome	Results	
										Change in NS	Effect
Correll et al., 2020 [159]	Double blinded RCT, phase III	LUM tosylate 40 mg/d, 60 mg/d	placebo	450	LUM tosylate 40 mg/d: 43.3 (10.1) LUM tosylate 60 mg/d: 42.4 (10.3) placebo: 41.4 (10.3)	Patients with acute relapse. Excluded first psychotic episode.	SZ	PANSS negative subscale total score	Primary outcome: change in PANSS total score. Secondary outcome: change in PANSS negative score	↔ vs. placebo	LUM tosylate 40 mg/d vs. placebo: PANSS negative LSMD −0.9, *p* = 0.36; LUM tosylate 60 mg/d vs. placebo: PANSS negative LSMD −1.4 *p* = 0.09
Lieberman et al., 2016 [160]	Double blinded RCT, phase II	LUM 60 tosylate mg/d, 120 mg/d Risperidone 4 mg/d,	placebo	335	LUM tosylate 60 mg/d: 38.3 (10.0) LUM tosylate 120 mg/d: 41.1 (8.9) RIS 4 mg/d: 40.7 (9.3) placebo: 40.5 (9.8)	Patients with acute relapse. Excluded first psychotic episode.	SZ	PANSS negative subscale total score	Primary Outcome: Change in PANSS total score Secondary outcome: change in PANSS negative score	↔ vs. placebo	LUM tosylate 60 mg/d vs. placebo: PANSS negative LSMD −0.9, *p* = 0.230; LUM tosylate 120 mg/d vs. placebo: PANSS negative LSMD + 0.7 *p* = 0.319; RIS 4 mg/d vs. placebo: PANSS negative LSMD −0.1 *p* = 0.914.

↔ similar results; CI: confidence interval; LSMD: least square mean difference; LUM: lumateperone; NS: negative symptoms; PANSS: Positive and Negative Symptoms Scale; RCT: randomized control trial; RIS: risperidone; SMD: standardized mean difference; SZ: schizophrenia; vs.: versus.

## Data Availability

Not applicable.
